# Assessing ASD in Adolescent Females with Anorexia Nervosa using Clinical and Developmental Measures: a Preliminary Investigation

**DOI:** 10.1007/s10802-017-0301-x

**Published:** 2017-04-17

**Authors:** Heather Westwood, William Mandy, Mima Simic, Kate Tchanturia

**Affiliations:** 1 Section of Eating Disorders, Psychological Medicine, Institute of Psychiatry, Psychology and Neuroscience, King’s College London, SE5 8AF, London, UK; 2 Clinical, Educational and Health Psychology, University College London, 1-19 Torrington Place, London, UK; 3 Child and Adolescent Eating Disorder Service, South London and Maudsley NHS Foundation Trust, London, UK; 4 Department of Psychology, Illia State University, Tbilisi, Georgia; 5 Psychological Medicine Clinical Academic Group, South London and Maudsley NHS Foundation Trust National Eating Disorders Service, London, UK

**Keywords:** Autism spectrum disorder, Anorexia nervosa, Comorbidity, Eating disorder

## Abstract

The aim of this study was to use standardised, clinical assessment tools to explore the presence of Autism Spectrum Disorder (ASD) symptoms in a sample of adolescent females with Anorexia Nervosa (AN), receiving either day-patient or inpatient treatment for their eating disorder and to determine whether any such symptoms were present during the early developmental period, a requirement for a diagnosis of ASD. Using a cross-sectional design, 40 females aged between 12 and 18 were recruited from inpatient and day-patient eating disorder services. All participants had a diagnosis of AN and were assessed for symptoms of ASD using the Autism Diagnostic Observation Schedule, 2nd edition (ADOS-2). If participants scored at or above clinical cut-off on the ADOS-2, their parents were asked to complete the Developmental, Dimensional and Diagnostic Interview, short version (3Di-sv). Of the 40 participants assessed, 21 scored above cut-off on the ADOS-2. When developmental history was obtained, only four participants scored above cut-off on all sub-scales of the 3Di-sv, thus meeting full research criteria for ASD. This study suggests that 10% of adolescents with AN from inpatient or day-patient settings may have diagnosable ASD, while a further 40% may show symptoms of ASD, which may arise from the ill-state of AN or are not supported by parental report.

## Introduction

Anorexia Nervosa (AN) is a severe eating disorder (ED) characterised by significantly low body weight; intense fear of gaining weight and undue influence of weight and shape on self-evaluation. Typically developing during adolescence, it effects more females than males (APA 2013). Autism Spectrum Disorder (ASD) on the other hand, is a pervasive developmental disorder with an onset in infancy, defined by difficulties with reciprocal social interaction and repetitive, restricted behaviours (RRBs). ASD is more prevalent in males than females (APA 2013).

Initial suggestion that some individuals with AN may have an underlying ASD (Gillberg and Rastam 1992) has led to prevalence estimates of ASD in ED populations, thought to be approximately 23% (for systematic review, see: Huke et al. 2013). This suggests a very substantial over-representation of ASD in EDs, given that ASD is thought to affect around 1% in the general population, about 0.2% of who are female (Baron-Cohen et al. 2009; Christensen et al. 2016). Studies assessing the prevalence of dimensionally-measured traits of ASD, assessed by self-report, also suggest an increased prevalence of difficulties associated with ASD, relative to healthy controls (Westwood et al. 2015).

Despite suggestion of increased prevalence of ASD in AN, few studies have focused on this co-morbidity in child and adolescent populations. Studies with young people have reported lower rates of ASD than in adult studies (Pooni et al. 2012), with only 4% of cases being assigned a possible ASD diagnosis (Rhind et al. 2014). These studies have relied on the use of tools assessing developmental history such as the Developmental, Dimensional and Diagnostic Interview (3Di; Skuse et al. 2004) or Development and Wellbeing Assessment (DAWBA; Goodman et al. 2000), which rely on the subjective insights of parents rather than providing an objective, observable measure of ASD symptoms. One recent study (Vagni et al. 2016) utilised the Ritvo Autism Asperger Diagnostic Scale Revised (RAADS-R; Ritvo et al. 2011) to assess the heterogeneity of ASD traits in ED populations aged 15 or over. Thirty three percent of participants were classified as having elevated ASD traits; however, despite the RAADS-R being used as a structured clinical interview in this study, it relies on the subjective insights of participants, particularly their ability to recall the presence of certain behaviours during childhood.

When diagnosing ASD, it is recommended that assessment include direct observation of core ASD symptoms as well as obtaining an early developmental history (NICE 2011). Postorino and colleagues (2017) used a well-validated, direct-observational measure of ASD, the Autism Diagnostic Observation Schedule, 2nd Edition (ADOS-2; Lord et al. 2012) with a group of adolescents with AN, receiving outpatient treatment. Only 10% of the participants met the required diagnostic cut-off on the ADOS-2 and the authors report that subsequent interview suggested that no individuals had a developmental history of ASD. Another study has used the ADOS-2 with a group of women with AN, recruited from specialist ED services (Westwood et al. 2017). Twenty-three percent of participants scored above cut-off on the ADOS-2 and elevated symptoms of ASD were associated with increased obsessive-compulsive symptoms and alexithymia. Despite efforts to obtain developmental history, a standardised diagnostic instrument was not used in either of these studies to obtain parental reports. To date, no studies have used the recommended combination of standardised parental and clinical measures to assess the presence of ASD in AN.

One of the difficulties associated with diagnosing ASD in young people with AN is that any symptoms that appear to be associated with ASD in this group may arise from the acutely ill-state of AN (Mandy and Tchanturia 2015). Individuals subjected to starvation, even without the psychological symptoms of an ED, display certain characteristics of ASD, including reduced cognitive flexibility and social motivation (Keys et al. 1950). Interestingly, individuals recovered from AN do not show some of the symptoms of ASD, such as difficulties with emotion expression, to the same extent as those who are acutely unwell (Dapelo et al. 2016). Younger people with AN appear to have less difficulty with central-coherence and set-shifting (Lang et al. 2015; Westwood et al. 2016) than adults, suggesting that these inefficiencies are exacerbated by the disorder. However, individuals with first-episode AN and those recovered from AN display difficulties with social functioning, suggesting that these difficulties may represent a stable trait associated with the disorder (Bentz et al. 2017). Thus, it may be that certain symptoms associated with ASD represent underlying factors in AN, whereas others are state-dependent.

One longitudinal cohort study assessed for ASD in 51 patients with adolescent onset-AN over an 18-year period. During this time, the prevalence of ASD rose from 7.8% at initial assessment (Gillberg and Rastam 1992) to 33% at follow-up (Anckarsater et al. 2012). While the reason for this apparent increase in prevalence remains unclear, it could be that reliance on clinical interviews such as the Structured Clinical Interview for DSM-IV (SCID; First 1997) without using a structured, validated parental tool to obtain a developmental history of ASD symptoms could lead to “false positive” diagnosis of ASD. If starvation or duration of AN increases the likelihood of exhibiting ASD symptoms then one would expect younger individuals, with a shorter illness duration to display fewer symptoms characteristic of ASD. Research utilising both clinical interview and developmental report with an adolescent sample would therefore be useful in clarifying the occurrence of ASD in ED populations.

Another issue in establishing whether ASD is truly over-represented in AN populations is that ASD presents differently in females, hindering diagnosis (Lai and Baron-Cohen 2015). Females with ASD tend to be diagnosed later than their male counter-parts (Begeer et al. 2013) and give accounts of “pretending to be normal”, in an attempt to fit in with their peers, resulting in professionals missing their diagnosis (Bargiela et al. 2016). Females with ASD can mask their difficulties (Lai et al. 2011), show higher social motivation (Head et al. 2014) and present with lower levels of RRBs (Frazier et al. 2014). These gender differences may help explain the disparity in ASD diagnosis. The gender ratio of 4:1 male to female (Fombonne 2009) is likely an exaggeration of the “true” gender ratio, which is difficult to determine without a completely objective test for ASD. Nevertheless, these gender differences have led to the proposal of a distinct female autism phenotype (Lai et al. 2015). In some women, EDs may also mask the presence of ASD, reflected in extreme rigidity in eating or obsession with calories or excessive exercise (Lai and Baron-Cohen 2015; Mandy and Tchanturia 2015). This highlights the need for robust, clinical assessment of ASD to be used within ED settings.

The few studies which have examined treatment outcome in individuals with AN and co-morbid ASD symptoms suggest that outcome is considerably worse for this group (Nielsen et al. 2015; Tchanturia et al. 2016). As it is not yet known whether this sub-group have “true” ASD or whether they present with symptoms characteristic of ASD due to their eating disorder, research which elucidates the relationship between the two disorders may be useful in informing future studies aiming to examine specific care pathways or treatment modifications for co-morbid AN and ASD.

The aim of the current study was to use a gold standard, observational measure alongside a developmental measures of ASD, namely the ADOS-2 and (Lord et al. 2012) and the 3Di-sv (Santosh et al. 2009) respectively, to determine the presence of ASD in a cross-sectional sample of adolescent females with AN, attending inpatient or day-patient treatment programmes.

## Method

### Participants

Forty females, aged 12 to 18 were recruited from either inpatient (*N* = 8) or day-patient (*N* = 32) ED services. These services were selected with the aim of recruiting individuals with more severe or treatment-resistant AN, resulting in a more homogenous sample. Sixty percent of young people approached about the study agreed to participate. Eighty-three percent (*N* = 33) of participants were White British, 7.5% (*N* = 3) described themselves as “White Other”. Two participants had dual heritage and the remaining two participants described their ethnicity as Indian and Black Caribbean, respectively. All new admissions to these services over a 16-month period were invited to participate. Inclusion criteria were: a primary diagnosis of AN and English speaking. The diagnosis of AN was made by a consultant psychiatrist within the respective ED services prior to inclusion in the study. The procedure of diagnosing AN differed by ED service but involved multi-disciplinary assessment using both DSM 5 and ICD 10 criteria. Sixty percent of young people approached about the study agreed to participate.

### Ethical Considerations

The study was approved by the National Research Ethics Service (14/LO/2131). Written informed consent was obtained from the parents of participants under the age of 16 or directly from participants aged 16 or over. Children under the age of 16 also gave their written assent to participate. All participants were paid £10 for their time.

### Procedure

Each participant completed the ADOS-2 in a single session with a trained, research-reliable examiner. If participants scored at or above clinical cut-off on the ADOS-2, their parents were contacted by telephone to complete the 3Di-sv. Ten percent of the ADOS-2 videos were watched by a second, independent researcher. Inter-rater reliability of coding was good, with 100% agreement on the overall ADOS-2 classification. In addition, each participant completed self-report questionnaires. Current weight and height were obtained from a clinician on the day of the assessment. Percentage of Ideal body weight (%IBW) was calculated, which expresses proportion of an individual’s optimal body weight, corrected for age and gender (Lock et al. 2010). Information on age that AN was first diagnosed was also obtained, which was used to calculate the duration of the ED (In years, calculated as age at ADOS-2 assessment minus age at which AN was diagnosed). Intelligence was also assessed to control for intellectual disability.

### Measures

#### Autism Diagnostic Observation Schedule, 2nd Edition (Lord et al. 2012)

The ADOS-2 is a standardised, semi-structured assessment for ASD and is the most widely-used and best validated direct observational measure of characteristics associated with ASD (NICE 2012). It focuses on the domains of social interaction, communication, play and imaginative use of materials. The ADOS-2 has four modules, one of which is selected dependent upon the participant’s expressive language abilities. Module 4 is designed for use with verbally fluent adolescents and adults and was used for all participants in the current study. The assessment is scored according to a standardised system and diagnostic algorithm, yielding an overall diagnosis.

The Module 4 algorithm (Hus and Lord 2014) has recently been revised to increase its comparability to other ADOS modules and to map on to DSM-5 diagnostic criteria. The revised algorithm consists of two sub-scores: Social Affect and RRBs. For the diagnostic cut-off, the sum of the two sub-scales is used. With respect to clinical ASD diagnosis, the revised Module 4 algorithm demonstrated high sensitivity (90.5%) and specificity (82.2%) (Hus and Lord 2014). The revised algorithm is superior to the original in that it has greater sensitivity and better reflects the current diagnostic criteria, particularly the symptoms displayed by females and adults with ASD (Pugliese et al. 2015) and was therefore used in this study.

#### Developmental, Dimensional and Diagnostic Interview, Short Version (3Di-Sv; Santosh et al. 2009)

The 3Di-sv is a brief, validated parental report, diagnostic measure of ASD with high test-retest and interrater reliability. It focuses on domains also addressed within the ADOS-2 including: language; social life; non-verbal communication; sharing and empathy; and play and interest. The algorithm yields three sub-scales: Reciprocal Social Interaction, Communication and Repetitive and Stereotyped Behaviour (RSB). The 3Di-sv demonstrates good sensitivity compared to the ADOS-2 confirmed clinical diagnosis for DSM-5 (Slappendel et al. 2016).

In this study, individuals were deemed to meet diagnostic criteria for ASD if they scored above cut-off on all three subscales. Due to some symptoms of ASD being commonly reported in females with AN, parents were specifically asked to focus on behaviours during infancy and childhood, prior to the onset of the ED.

#### Eating Disorders Examination Questionnaire (EDE-Q; Fairburn and Beglin 1994)

The EDE-Q is a well validated self-report measure of the severity of the characteristic psychopathology of EDs. It contains 36 items which ask respondents to rate how often they have engaged in certain eating disordered behaviours or held eating disordered concerns over the past 28 days. The scores result in a ‘global’, or total score, with higher scores indicative of greater severity. In this study, Cronbach’s alpha was 0.86, indicating high internal reliability.

#### Mood and Feelings Questionnaire (MFQ; Costello and Angold 1988)

The MFQ is a 33-item self-report measure assessing symptoms of major depressive disorder in young people. The MFQ has moderate to high criterion validity for discriminating major depressive episodes from other mood disorders in adolescents, diverse in demographic and clinical characteristics (Daviss et al. 2006). Cronbach’s alpha was 0.95 in the current study.

#### The Obsessive Compulsive Inventory – Revised (OCI-R; Foa et al. 2002)

The OCI-R is an 18 item self-report scale with a five point Likert scale format. It consists of six subscales: Checking, washing, obsessing, neutralising, ordering and hoarding, yielding both a total and total and individual subscale scores. The OCI-R has good to excellent internal reliability among both clinically anxious and non-anxious participants and good test-retest reliability. It is recommended for the assessment of obsessive-compulsive symptoms in individuals with AN (Roberts et al. 2011). In this study, Cronbach’s alpha was 0.94. The OCI-R was included in the study as symptoms of ASD co-occur at high rates in young people with OCD (Griffiths et al. 2017) and thus could confound the relationship between ASD and AN.

#### Wechsler Abbreviated Scale of Intelligence, 2nd Edition (WASI-II; Wechsler 2011)

The WASI-II is an individually administered assessment of intelligence, suitable for individuals aged 6–90 years old. It provides scores that estimate intelligence in verbal and perceptual reasoning as well as full-scale IQ. The WASI-II includes four sub-tests: Matrix Reasoning, Block Design, Vocabulary and Similarities.

### Statistical Analysis

To examine the association between symptoms of ASD and both psychiatric symptoms (ED psychopathology, depression and obsessive compulsive symptoms) and demographics, participants were split into two groups: those who scored at or above clinical cut-off on the ADOS-2 and those who scored below. Independent sample t-tests were conducted to explore group differences. Dependent variables were checked for normality visually using histograms and statistically using the Shapiro-Wilk’s test. If data were not normally distributed, transformations were applied where possible and are reported with the results (EDE-Q). If normality could not be achieved through data transformation, non-parametric Mann-Whitney U tests were used instead (duration of AN). An alpha value of 0.05 was applied for all tests of statistical significance. Data were analysed using the statistical package IBM SPSS version 22.00. Cohen’s D effect sizes are provided for all comparisons using the following interpretation: small (0.20), medium (0.50) and large (0.8) (Cohen 1988).

## Results

### Presence of Symptoms Associated with ASD

Of the 40 participants, 21 (52.5%) scored at or above clinical cut-off on the revised ADOS-2 algorithm (Hus and Lord 2014). Of those who scored at or above cut-off, 12 displayed RRBs, which despite being part of the diagnostic criteria for ASD, are not required as part of the diagnostic algorithm of the ADOS-2. A comparison of clinical and demographic variables between participants who scored at or above and below cut-off on the ADOS-2 is displayed in Table 1. The groups did not differ significantly on any measure. No participant exhibited signs of intellectual disability (IQ < 75).

**Table 1:** Mean and Standard Deviations with independent t-test results for clinical and demographic variables between participants scoring at/above and below clinical cut-off on the ADOS-2

	Below cut-off *N* = 19	Above cut-off *N* = 21	*p*	Effect size
Recruitment site	Inpatient =3 Outpatient =16	Inpatient =5 Outpatient =16		
Age	15.58(1.35)	14.86(1.62)	0.136	*d* = 0.58
Age of onset of AN	14.58(1.39)	13.62(1.88)	0.077	*d* = 0.58
Duration of AN*	1.00(1.00)	1.00(1.00)	0.789	*r* = 0.05
%IBW	87.18(8.46)	88.51(7.19)	0.599	*d* = 0.17
EDE-Q Global score	3.95(1.50)	3.80(1.38)		
Transformed EDE-Q Global score	1.53(0.44)	1.58(0.41)	0.707	*d* = 0.12
MFQ score	70.13(16.10)	71.11(14.24)	0.851	*d* = 0.07
OCI-R score	27.73(17.98)	33.74(18.05)	0.342	*d* = 0.34
WASI-II FSIQ	109.26(12.19)	108.15(16.16)	0.810	*d =* 0.08
% binge-purge subtype % Atypical AN	5.3 10.5	4.8 14.3		

*EDE-Q* Eating Disorder Examination Questionnaire, *WASI-II FSIQ* Wechsler Abbreviated Scale of Intelligence Full-scale IQ, *MFQ* Mood and Feelings Questionnaire, *OCI-R* Obsessive Compulsive Inventory – Revised, *%IBW* percentage of ideal body weight

*Mann-Whitney U test, medians and interquartile ranges are reported along with Pearson’s *r* effect size, 0.05 indicates a negligible effect

Of the 21 young-people who scored at or above cut-off on the ADOS-2, four also scored above cut-off on all sub-scales of the 3Di-sv. These cases were discussed with the multi-disciplinary team involved in each participant’s care and a best-estimate diagnosis of ASD was given in each of the four cases who scored above cut-off on the measures. This resulted in 10% of the entire sample meeting diagnostic criteria for ASD. In addition, one participant scored above cut-off on the Reciprocal Social Interaction subscale but not on Communication or RSBs on the 3Di-sv. Another three participants displayed some behaviour associated with ASD in all domains but did not reach clinical cut-off, while the parents of all other participants (*N* = 13) reported no evidence of behaviours associated with ASD prior to the onset of their daughter’s ED.

### Qualitative Description of Participants who met Diagnostic Criteria for ASD

All four of the participants who scored above cut-off on both the ADOS-2 and 3Di-sv were suspected of having ASD by their clinical teams prior to participating in this study. All four participants had been diagnosed with restrictive AN for less than two years at the time of assessment. The parents of all four young-people reported believing that their daughters may have had ASD or another developmental disorder but none had ever been assessed. Two participants had a brother with a diagnosis of ASD.

#### Other co-Morbidities

One participant had a diagnosis of selective mutism, although she did speak during the ADOS-2. No other co-morbidities were reported for the other participants.

#### Social Communication and Interaction

During the ADOS-2, all four participants had difficulty with conversation and use of descriptive and emphatic gesture was either absent or extremely limited. All used poorly modulated eye contact and did not direct facial expressions towards the examiner. Lack of insight into the nature of typical social relationships was displayed by all participants and the quality of social overtures was reduced or there were no social overtures of any kind. The overall quality of social response was reduced, as was the quality of rapport with the examiner. Three of the four participants did not spontaneously ask about the experiences of the examiner and had difficulty describing events outside of the immediate context. Three participants expressed little shared enjoyment in the interaction and did not comment on other’s emotions. There was a lack of reciprocal social interaction and maintenance of attention.

From the 3Di-sv, three of the four mothers of the young people reported delays in their daughter’s language acquisition, being diagnosed with some form of language disorder as children. Two of the four made errors of pronoun, e.g., saying “she” instead of “he”. They were all reportedly reluctant to engage in “small talk” and had difficulty forming and maintaining friendships, finding it difficult to understand social norms. Regarding non-verbal communication, all reported unusual eye contact. They all had difficulty understanding how others were feeling from their facial expression or tone of voice and all found it hard to express their own emotions.

#### Restricted, Repetitive Patterns of Behaviour

During the ADOS-2, all four participants displayed speech abnormalities associated with ASD e.g., speaking with a flat intonation. Only two participants displayed evidence of stereotyped behaviours or restricted interests, e.g., repeatedly referring to a school teacher or having an overriding interest in West End musicals. From the 3Di-sv, it became apparent that all four had very specific or overriding interests, including figure skating and boy bands. They all played in a rather formal way as children, rather than engaging in imaginative play. Three displayed some infantile-like interest in taste or smell, e.g., licking objects such as stones and exhibited complex mannerisms or stereotypy such as spinning around or hand flapping.

## Discussion

This is the first study to utilise standard, clinical assessment tools: the ADOS-2 and 3Di-sv, to assess ASD in adolescents with a diagnosis of AN, admitted to either day-patient or inpatient treatment programmes. More than half (52.5%) scored above the clinical cut-off on the ADOS-2, however, when developmental history was obtained, only 10% met diagnostic criteria for ASD. Therefore, while a high proportion of adolescent females display symptoms of ASD when they have AN, in most cases, these symptoms may arise from the ill-state associated with AN. Alternatively, parents may not recognise or may under-report symptoms of ASD in their daughters.

The estimate from the current study that 10% of individuals meet research criteria for ASD is higher than in other studies with young people (Postorino et al. 2017; Rhind et al. 2014). Despite Postorino et al. (2017) also using the ADOS-2 with adolescents with AN, only 10% of their sample met diagnostic cut-off for ASD. As the participants recruited by Postorino and colleagues were receiving outpatient treatment, it could be that they represented a less severe group of patients than those recruited in this study. A recent study (Stewart et al. 2017) suggests that the presence of ASD traits is associated with greater need for treatment augmentation. Thus, individuals recruited from inpatient or intensive day-treatment programmes may be more likely to exhibit symptoms of ASD than individuals receiving lower-intensity care. One other study (Bentz et al. 2017) using the ADOS-2 with young people with first-episode AN found that 16% of individuals scored above the suggested cut-off. The findings from these studies and the current study suggest the presence of symptoms of ASD in a significant proportion of young females with AN. However, future research is needed to confirm whether such symptoms are present prior to the onset of the ED.

The findings from this study support the notion that ASD is over-represented in AN populations, in line with previous adult studies (Huke et al. 2013). However, as it is the case that individuals with ASD are also at increased risk of developing other psychiatric disorders e.g., anxiety disorders (van Steensel et al. 2011), depression (Sterling et al. 2008) and Schizophrenia (King and Lord 2011), ASD may not specifically be associated with the development of EDs but rather psychopathology in general. Further research comparing the presence of ASD in females across disorders would therefore be beneficial. However, as the high and low scoring ASD symptom groups in this study did not differ on measures of depression or obsessive-compulsive symptom, it does not appear as if these psychiatric symptoms influenced the relationship between ASD and AN in this cohort, contrary to what was found in one previous adult study (Westwood et al. 2017).

One possible explanation for the discrepancy between the finding from this study and those from previous studies is our use of both observational and developmental measures. Females with ASD are underrepresented at the higher end of the IQ distribution (Lai et al. 2012) and often present with fewer social difficulties than their male counterparts (Head et al. 2014). Symptoms of ASD in females may also be erroneously interpreted as gender-typical behaviour, e.g., interest in dolls being interpreted as pretend play (Halladay et al. 2015) or mistaking an overriding interest as a normal teenage fascination (Attwood 2007). Females with ASD are known to be able to camouflage some of their difficulties (Bargiela et al. 2016; Head et al. 2014; Lai et al. 2011) and these factors, along with the public misconception of ASD as a “male disorder” may have prevented parents in previous studies from recognising symptoms of ASD in their daughters. This may have also accounted for the low concordance rate between the ADOS-2 and 3Di-sv in the current study as parents may never have considered the possibility of ASD in their child, leading to inaccurate retrospective reports.

Use of other parental-report measures of ASD such as the Autism Diagnostic Schedule-Revised (ADI-R; Lord et al. 1994) alongside the ADOS-2 increases the sensitivity and specificity of detecting ASD in young people (Risi et al. 2006). While studies assessing agreement of the ADOS and ADI-R to diagnose children with ASD suggest low to good levels of agreement between the two measures (e.g., Gray et al. 2008; Le Couteur et al. 2008; Ventola et al. 2006), studies of concordance have not extended to cohorts experiencing mental health problems. It is therefore not possible to determine whether issues with concordance between parental report and clinical observation are confined to the 3Di or whether they extend to other standardised diagnostic instruments. This line of research would benefit from further studies utilising both the ADOS-2 and parental measures with an eating disorder cohort. However, the 3Di-sv has demonstrated good sensitivity and fit against DSM-5 criteria for ASD (Slappendel et al. 2016) and has demonstrated high agreement with the ADI-R (Santosh et al. 2009), thus offering a quick and cost-effective alternative to more lengthy, expensive measures.

While the male bias in ASD diagnosis may account for some of the discrepancy between the ADOS-2 and 3Di-sv, this study also supports the notion that while individuals with AN display characteristics associated with ASD, these symptoms may represent a superficial similarity between the two disorders rather than a co-morbid presentation. Similarities have been observed in several domains including neuropsychological and socio-emotional processing (Doris et al. 2014; Oldershaw et al. 2011; Westwood et al. 2016). However, similarities in these areas are also observed in other psychiatric disorders, such as schizophrenia (Konstantareas and Hewitt 2001). While ASD is known to be over-represented in schizophrenia, the two disorders may also be characterised by traits which mimic one another, such as difficulties with social interaction and lack of emotional response, resulting in a superficial similarity leading to misdiagnosed co-morbidity (Chisholm et al. 2015); this could also explain part of the relationship between ASD and AN.

Alternatively, it could be that certain characteristics, such as difficulties with social cognitive associated with ASD are also present in AN (Bentz et al. 2017) while the prevalence of full ASD in AN remains relatively low. This overlap in symptoms makes recognising and diagnosing ASD difficult, particularly as other symptoms of ASD such as extreme rigidity may be attributed to AN if manifested in preoccupation with calories or exercise (Lai and Baron-Cohen 2015). This issue of differential diagnosis highlights the importance of combing both standardised assessment with best clinical judgment in the diagnosis of ASD in this cohort. It may also explain why only four participants met full diagnostic criteria for ASD, despite so many scoring about cut-off on the ADOS-2.

Disentangling social and communication problems associated with ASD from other complex clinical presentations in adulthood is particularly challenging (Bastiaansen et al. 2011). This may be exacerbated in individuals with AN, who often present with other co-morbidities including anxiety and depression (Blinder et al. 2006), which could in turn cause social interaction difficulties assessed for in the ADOS-2 to appear to be associated with ASD. This may also account for the high proportion of females with AN who score above cut-off on self-report measures of symptoms of ASD, such as the AQ (Westwood et al. 2015).

### Limitations

Not controlling for social anxiety is a limitation of this study, given that individuals with AN often present with co-morbid social anxiety (Swinbourne and Touyz 2007), which could lead to high scores on the ADOS-2. While effort was made to account for developmental history of ASD, it is still not possible to determine whether ASD symptoms were present prior to the onset of AN in all cases. Longitudinal cohort studies which identify at-risk populations, e.g., females diagnosed with high-functioning ASD and then determine the proportion who develop EDs would further elucidate the relationship between the disorders.

As all the participants in the current study were recruited from either day-patient or inpatient treatment programmes, they likely represent a more severe sub-group of patients with AN i.e., who do not respond to outpatient treatment, and may be more likely to display symptoms of ASD than individuals recruited from outpatient programmes. The cross-sectional nature of the study and exclusive inclusion of participants from either inpatient or day-patient services therefore limits generalisability of the study findings. The fact that all participants were in the acute phase of AN e.g., with current ED psychopathology, may have exacerbated the presence of ASD symptoms. It is also possible that our sample was biased by individuals who were curious about the possibility of having a diagnosis of ASD being more likely to participate. Data on reasons for declining to participate were not collected but may be important to consider in future research. Future longitudinal research examining the relationship between severity of AN and ASD would therefore be useful to build on the small sample size and preliminary findings presented here.

## Conclusion and Implications

The high proportion of females scoring above cut-off on the ADOS-2 who have no developmental history of ASD highlights the need for rigorous assessment of ASD within ED populations. Relying on self-report or clinical interview alone could lead to misdiagnosis of ASD. While the limited existing research suggests worse outcomes in individuals with AN and ASD symptoms, recovery from AN could lead to a reduction in these symptoms, highlighting the need for longitudinal studies examining treatment outcome in individuals who have received a rigorous diagnosis of ASD. On the other hand, if females with ASD really are at an increased risk of developing EDs then interventions specifically targeted at this group could be beneficial.

The current study showed no difference in high and low scores on the ADOS-2 in terms of clinical characteristics, i.e., ED symptoms, depression or OCD, suggesting that symptoms of ASD may not arise purely because of the ill-state of the ED. it could be that other problems associated with AN such as social anxiety, which was not assessed in this study, may cause false-positive results on the ADOS-2. It is important for clinicians to consider alternative reasons for why individuals may score above clinical cut-off on ASD measures and to consider the possibility of addressing these symptoms within treatment.
